# DNA methylation and immune regulation in osteoporosis: emerging epigenetic targets for drug discovery

**DOI:** 10.3389/fphar.2025.1688305

**Published:** 2025-11-07

**Authors:** Yingli Yang, Yao Yao, Xiaoyu Cai, Yaqi Tao, Zhengbing Zhuge, Jianhong Zhou, Caihong Zheng

**Affiliations:** 1 Department of Pharmacy, Women’s Hospital School of Medicine, Zhejiang University, Hangzhou, China; 2 Department of Pharmacy, Affiliated Hangzhou First People’s Hospital, School Of Medicine, Westlake University, Hangzhou, China; 3 Department of Gynecology, Women’s Hospital, Zhejiang University School of Medicine, Hangzhou, Zhejiang, China

**Keywords:** osteoporosis, DNA methylation, osteoblasts, osteoclasts, immune regulation

## Abstract

Osteoporosis (OP) is a complex skeletal disease characterized by the disruption of bone homeostasis, with immune dysregulation identified as a significant pathogenic cause. The interaction between immune cells and bone cells within the bone marrow microenvironment affects osteoclast (OC) activation and osteoblast (OB) function through cytokine networks, including RANKL/OPG and Wnt signaling. Aberrant DNA methylation, a significant epigenetic change, influences osteoporosis by regulating the expression of genes associated with bone metabolism (e.g., RUNX2, NFATc1, SOST) and modifying immune cell activities, thereby facilitating inflammatory bone loss. Increased DNA methyltransferase (DNMT) activity has been associated with osteoimmune dysregulation, oxidative stress, and heightened bone resorption. Inhibiting DNMT inhibitors (DNMTi) has shown effectiveness in preclinical animals by reversing abnormal methylation patterns and restoring bone mass. Additionally, DNA methylation profiles generated from peripheral blood exhibit significant concordance with bone tissue methylation, presenting prospective non-invasive biomarkers for OP diagnosis, prognosis, and therapy monitoring of OP. Incorporating epigenetic profiling into clinical practice could facilitate precision medicine strategies for OP, combining immune regulation with targeted DNA methylation therapy. This study emphasizes the relationship between DNA methylation and osteoimmunity, delineating innovative treatment targets and biomarker prospects to enhance OP therapy.

## 1 Introduction

Osteoporosis (OP) is a systemic skeletal disorder characterized by diminished bone mass and progressive degradation of bone microarchitecture, leading to increased bone fragility and a significantly heightened risk of fractures. Epidemiological data reveal that over 200 million individuals globally are afflicted by this condition. The incidence of OP escalates notably with age, particularly among postmenopausal women and older adults (Li et al., 2024). The most clinically significant outcome of OP is fragility fractures, which primarily occur at the spine, hip, and wrist. As bone structural damage worsens, clinical symptoms such as joint pain, weight loss, and compromised respiratory function may appear (Ali and Kim, 2025).

The etiology of OP requires intricate regulation by various factors, including age, hormone levels, inflammation, immunology, and more elements. The conventional viewpoint asserts that OP results from a disparity between osteoclast (OC)-mediated bone resorption and osteoblast (OB)-mediated bone production, representing the basic pathological mechanism of the disease (Luo et al., 2025). Nevertheless, the skeletal system operates in conjunction with other systems. Arron presented the notion of “osteoimmunology”, emphasizing the intricate bidirectional regulatory interactions between the skeletal and immune systems (Arron and Choi, 2000). Srivastava, R. K. et al. Clarified the functions of distinct T lymphocyte subsets in the pathophysiology of OP, leading to the emergence of a new biological domain called “immuno-osteoporosis” (Srivastava et al., 2018). Thus, the involvement of the immune system in the pathogenesis of OP has attracted considerable scientific attention. A two-sample Mendelian randomization (MR) study identified significant associations between 32 immune cell phenotypes and 38 inflammatory cytokines with OP (Kuang et al., 2025). Research demonstrates that many immune cells (such as T cells, B cells, macrophages, etc.) and cytokines (such as RANKL, TNF-α, IL-17, etc.) can directly or indirectly influence the differentiation and function of OB and OC. Recent research utilizing single-cell RNA sequencing technology has identified that CCL4+ NKT and CXCL8 neutrophil subsets facilitate the advancement of OP by orchestrating an inflammatory milieu that disturbs bone homeostasis. Furthermore, the MNDA + macrophage subpopulation was identified as promoting OC development, hence intensifying OP (Yang et al., 2024). Overall, these data underscore a novel domain in bone immunology, elucidating a complicated inter-regulatory relationship between the immune system and the skeletal system in OP.

DNA methylation significantly influences immune regulation and bone metabolism. Under standard physiological conditions, DNA methylation generally inhibits gene transcription, whereas demethylation facilitates gene expression. Disruption of this balance can result in immune system diseases and irregular bone metabolism. Recent data indicate that DNA methylation may be linked to age-related diseases and skeletal biology (Curtis et al., 2022). Genome-wide association analysis indicates a notable trend of hypomethylation across the genome in trabecular bone tissue of OP patients, with critical bone metabolism genes (including RUNX2, SOST, and OPG) displaying distinct methylation modifications (Delgado-Calle et al., 2013). Furthermore, aberrant methylation of the Wnt pathway diminishes osteogenic activity, and hypomethylation of the RANKL promoter can augment osteoclast function (Husain and Jeffries, 2017). Zhang et al. established that DNMT3A-induced hypermethylation of FoxO3 results in bone marrow mononuclear macrophage oxidative stress imbalance and increased OC production, ultimately leading to OP (Zhang et al., 2025). These investigations further confirm that DNA methylation involved in the pathophysiology of OP by modulating the interplay between immune response and bone metabolism.

The primary main strategies for managing OP are physical exercise, a balanced diet, and pharmacological treatment. Drug therapy primarily includes agents that prevent bone resorption (such as bisphosphonates, RANKL inhibitors) and drugs that promote bone formation (such as parathyroid hormone analogues) (Brent, 2023; LeBoff et al., 2022). Nonetheless, current therapy modalities exhibit issues such as side effects and insufficient long-term efficacy, and they fail to fully reverse the pathological process of osteoporosis. The reversible nature of DNA methylation allows for selective modulation of its status, presenting a unique strategy for OP. Among them, the DNA methyltransferase (DNMT) inhibitor SGI-1027 can reverse the hypermethylation state of the GPX4 promoter in OP animal models, restore its expression, and reduce ferroptosis of OB (Ruan et al., 2024). Another DNMT inhibitor, 5-aza-2′-deoxycytidine (5-AZA-Dc), can significantly ameliorate osteogenic impairment in disuse OP (Li et al., 2018). Therefore, this review seeks to thoroughly examine DNA methylation and immune regulation in OP, providing a theoretical basis for the development of anti-osteoporosis pharmaceuticals that target epigenetic factors.

## 2 The mechanism of immune regulation in OP

**FIGURE 1:**
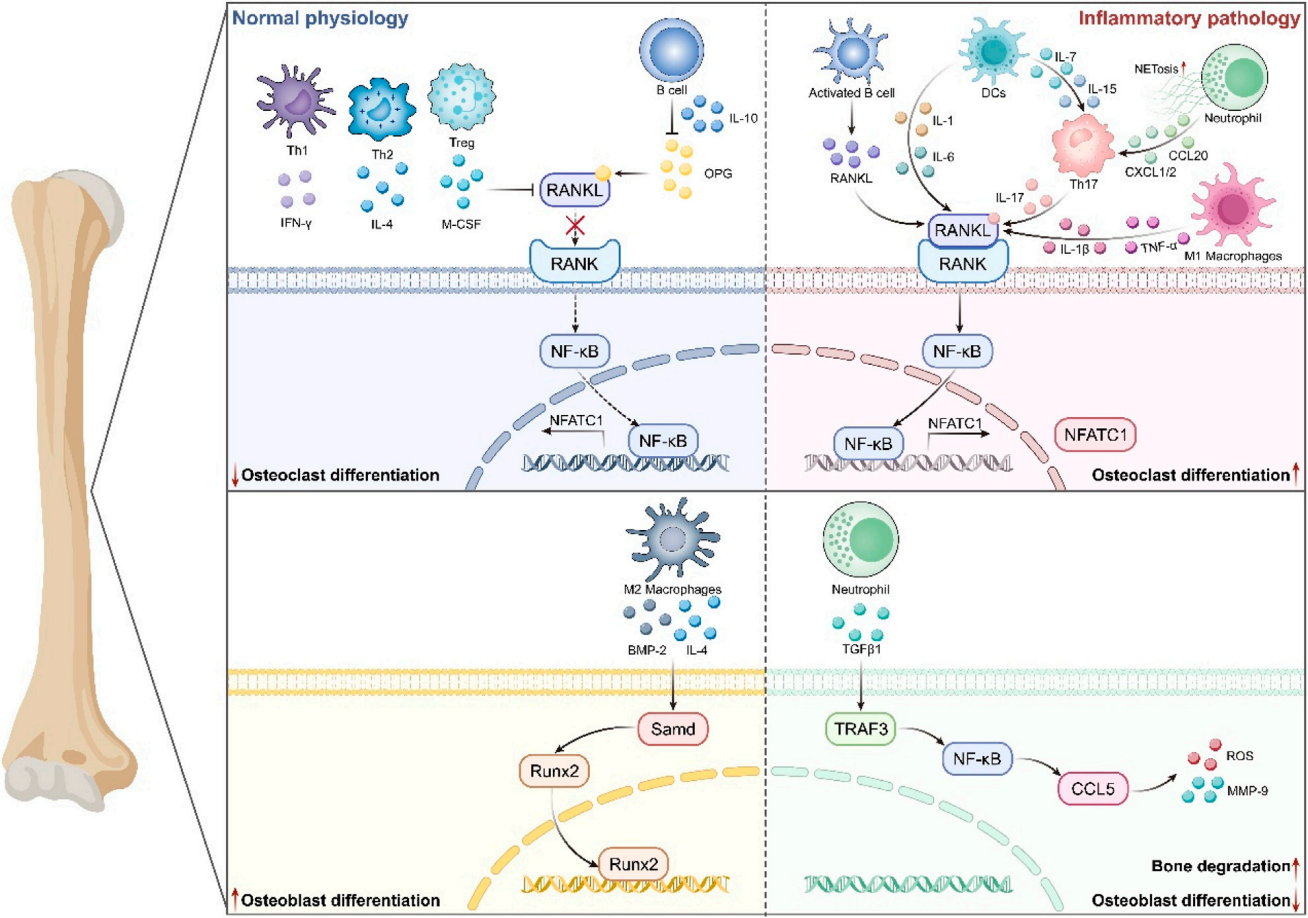
The mechanism of the immune system in Osteoporosis. Immune cells influence the process of bone formation and bone resorption by generating various cytokines and interacting with other immune cells. It mainly includes that under normal physiology, Th1/Th2/Treg and B cells regulate pathways such as RANKL/RANK and NF-κB by mediating various cytokines, thus inhibiting osteoclast differentiation. Also, M2-type macrophages participate in secreting IL-4 and BMP-2 to promote the expression of Runx2 and the differentiation of osteoblasts. In an inflammatory environment, activated B cells, Th17, DCs, neutrophils, etc., enhance the RANKL/RANK pathway through cytokines like IL-1/6/17, driving osteoclast differentiation. Meanwhile, neutrophils inhibit osteoblast differentiation via pathways such as TGFβ1 and NF-κB, ultimately leading to the pathological mechanism of bone degradation.

The immune system is essential for preserving bone homeostasis. On one hand, bone cells and immune cells coexist in the same bone marrow microenvironment, facilitating the exchange of various cytokines and signaling molecules. Conversely, immune cells are involved in bone metabolism by modulating essential processes, including inflammatory responses and bone resorption/formation. This signifies that the immune system is crucial to the progression of OP. A multitude of immune cells have been demonstrated to directly or indirectly affect bone cells through cytokines such as OPG/RANKL, IL-6, and TNF-α, along with other immune-derived substances. Therefore, this review will examine the influence of the innate and the adaptive immune systems on bone metabolism mechanisms pathways, serving as a reference for research in OP (Figure 1).

### 2.1 Adaptive immune system

T cells are the primary cells that govern cell-mediated immunity. In the adaptive immune system, T cells differentiate and secrete numerous cytokines that modulate bone metabolism. Activated T cells secrete RANKL, which interacts with RANK on the surface of OCs, facilitating their development and augmenting bone resorption activity. Research indicates that GIOP cannot be induced in SCID mice, which are devoid of T cells, but the condition can be reinstated through the adoptive transfer of spleen T cells from wild-type mice. Mechanistically, activated T cells highly express RANKL, leading to OP (Song et al., 2020). Helper T cells (Th), including Th1 and Th2 subsets, release characteristic cytokines such as IFN-γ and IL-4, both of which obstruct RANKL signaling and inhibit OC formation. Th17 cells secrete various cytokines, such as IL-17, IL-21, and IL-22. Furthermore, numerous studies indicate that Th17 cells are significantly implicated in various inflammatory diseases, including OP, rheumatoid arthritis (RA), and inflammatory bowel disease (IBD) (Lu et al., 2025). Treg cells directly inhibit the generation of OCs by suppressing the production of RANKL and M-CSF. Subsequent study reveals that reduced Treg cell numbers and functions, along with heightened Th17 cell activity, result in an imbalance between Tregs and Th17 cells culminating in exacerbated inflammatory responses and irregularl bone metabolism (Liu et al., 2024b). Moreove, a T cell-depleting mesoporous silica nanoparticle has been shown to ameliorate bone loss in ovariectomized (OVX) models by diminishing activated T cells and modifying the Treg/Th17 balance (Yang et al., 2021). All data indicates that T cells, as immune cells, can regulate bone homeostasis by direct interaction with bone tissue cells or via paracrine pathways, which is closely associated with OP.

B cells, principally engaged in humoral immunity, significantly influence bone metabolism. Under healthy settings, they release OPG, which inhibits OC activity by blocking RANKL-RANK binding. However, in postmenopausal osteoporosis (PMO), activated B cells enhance RANKL production, facilitating OC formation. Similarly, we also identified the B cell subsets that produce RANKL in RA (Meednu et al., 2016). Consequently, rituximab, as an anti-CD20 antibody that depletes B cells, is applicable in the treatment of RA. Genetic investigations in RANKL^−/−^ and OPG^−/−^ mice further substantiate the association between B cell dysfunction and abnormal bone remodeling (Sanz et al., 2007). Another clinical study has shown that bone resorption in HIV patients significantly increases. Among them, the ratio of RANKL/OPG in B cells is elevated, indicating that rapid bone loss is associated with B cell dysfunction (Titanji, 2017). Regulatory B cells (Bregs) suppress the differentiation and functional activity of OCs through the secretion of IL-10. Notably, PMO patients exhibit a substantial reduction in Bregs and their capacity to release the IL-10 cytokine, consequently worsening inflammatory bone loss. Recent findings indicate that *Bacillus* coagulans supplementation may attenuate PMO-related bone loss by influencing the Breg-Treg-Th17 axis and gut microbiota (Sapra et al., 2025). In summary, B cells play a crucial role in the immune system of OP.

### 2.2 Innate immune system

Macrophages, as a crucial cell type in the osteoimmune milieu, experience phenotypic polarization influenced by the regulation of the body’s microenvironment. It can differentiate into two subtypes: conventionally activated (M1), which possesses pro-inflammatory characteristics and alternatively activated (M2), which exhibits anti-inflammatory capabilities (Muñoz et al., 2020). M1 macrophages can stimulate the NF-κB/RANKL signaling pathway through the secretion of TNF-α and IL-1β, thereby augmenting the activity of OCs. In addition, M1 macrophages serve as progenitors to OCs. Consequently, an increase in the number of M1 macrophages will further accelerate the bone resorption process (Yan et al., 2024). In the OVX mouse model, M1 macrophage exosomes (M1-exos) can exacerbate bone loss in OP by downregulating DUSP1 and activating the JNK signaling pathway (Yu et al., 2021a). M2 macrophages emit not only the anti-inflammatory cytokine IL-4 but also osteogenic factors such as BMP-2. Among them, BMP-2 can mediate the translocation of Runx2 into the nucleus through Smad, hence enhancing the differentiation of OBs (Shen et al., 2021). However, in the absence of RANKL, IL-4 can impede TNF-α-induced OC differentiation via the STAT6 mechanism. Recent studies have shown that Bilobalide can facilitate the polarization of macrophages toward the M2 phenotype, suppress OC differentiation by activating the SIRT3/NF-κB signaling pathway, and subsequently enhance bone mineral density in OP mouse models (Qin et al., 2024). The imbalance of macrophage polarization can cause immune dysregulation, leading to inflammatory activation and the production of several inflammatory agents that impact bone metabolism, ultimately culminating bone metabolic diseases such as RA and OP (Liu et al., 2024a). Consequently, modulating the M1/M2 ratio and promoting M2 polarization are anticipated to emerge as a novel treatment approach for enhancing OP.

Neutrophils, the predominant subset of white blood cells, are integral to the various regulatory processes of OP, mostly by exacerbating inflammation and disrupting bone metabolism (Zhang et al., 2024). On one hand, neutrophils can specifically recruit Th17 cells by secreting chemokines such as CCL20 and CXCL1/2, thereby increasing the activation of the IL-17/RANKL pathway, which directly exacerbates OC differentiation. Research indicates that the percentage of RANKL + neutrophils in the bone marrow of RA patients is directly linked with the extent of bone erosion, implying their possible direct role in inflammatory bone deterioration (Hajishengallis et al., 2016). In the latest research, we found that NETosis of neutrophils in OVX mice is significantly activated, inducing M1 polarization of macrophages and the production of osteoclastogenic factors via the cGAS-STING/AKT2 pathway (Guo et al., 2025). Conversely, in age-related OP, TGFβ1 facilitates neutrophil infiltration through the TRAF3/NF-κB/CCL5 pathway, establishing a CCL5-CCR5 positive feedback loop that induces the release of ROS and MMP-9, thereby impeding OB differentiation and expediting bone deterioration. This process elucidates the correlation between persistent low-grade inflammation and diminished bone production noted in age-related OP (Li et al., 2023). In conclusion, neutrophils have a role in the pathogenesis of OP through many mechanisms, offering a new basis for targeted immunological treatments.

Dendritic cells (DCs), as pivotal antigen-presenting cells, play a central role in linking innate and adaptive immune responses and driving final immune effects by mediating the activation of naïve T cells. In the regulation of bone metabolism, DCs are produced in the bone marrow and secrete multiple pro-inflammatory substances (such as IL-1, IL-6, TNF-α), which directly facilitate the activation of OCs through the activation of the RANK/RANKL signaling pathway. Furthermore, immature DCs are essential precursor cells for OC in maintaining bone homeostasis and mediating inflammatory responses (Puchner et al., 2024). Their dysfunction, facilitated by the transduction of signaling complexes such as RANK-RANKL-OPG-TRAF6, has been substantiated as closely linked to bone-destructive diseases like RA, OP, and periodontitis (Liu and Teng, 2023). Furthermore, DCs activate T cells, prompting them to release RANKL, which subsequently enhances the bone resorption activity of OB. Studies demonstrate that in the context of estrogen shortage, DCs exhibit elevated expression of IL-7 and IL-15, which collectively stimulate memory T cells to secrete IL-17A and TNF-α, ultimately enhancing OC generation (Cline-Smith et al., 2020). Recent advances in defining the cellular and molecular axes of immune–bone crosstalk, particularly in the context of osteoporosis, are outlined in Table 1.

**TABLE 1:** Immune–bone crosstalk (recent highlights) relevant to osteoporosis.

Immune subset/axis	Key mechanism	Disease/model and outcome	Refs
Neutrophils → macrophages/OCs (NETosis)	NETosis activates cGAS–STING–AKT2 signaling, amplifying osteoclastogenesis	Postmenopausal/experimental osteoporosis; ↑ bone resorption	Guo et al. (2025)
TGFβ1^+^CCR5^+^ neutrophils (aging)	TGFβ1 drives CCR5^+^ neutrophil subset that promotes OC activity	Age-related OP in male mice; causative neutrophil subset	Li et al. (2023)
Dendritic cells → osteoclasts	Bona fide DCs serve as pivotal osteoclast precursors	Homeostasis and inflammatory bone loss; OC lineage clarified	Puchner et al. (2024)
Memory T cells (post-OVX)	OVX triggers chronic low-grade inflammation mediated by memory T cells	Mouse; inflammation-driven bone loss after OVX	Cline-Smith et al. (2020)
T cells in GIOP	T cells required for glucocorticoid-induced osteoporosis; adoptive transfer restores phenotype	GIOP models; T-cell dependence established	Song et al. (2020)
B cells → RANKL → OCs	Switched memory B cells produce RANKL and promote osteoclastogenesis	RA; link to bone erosion via B-cell RANKL	Meednu et al. (2016)
Neutrophils in inflammatory bone loss	Regulatory and destructive roles of neutrophils in bone loss (e.g., periodontitis)	Review synthesizing neutrophil pathways in bone loss	Hajishengallis et al. (2016)
Gut–Th17 trafficking after OVX	OVX expands intestinal Th17/TNF^+^ T cells; gut–BM trafficking drives bone loss; blockade prevents loss	OVX mice; mechanism-based prevention demonstrated	Yu et al. (2021b)
Systemic inflammaging methylation signature	Blood DNA-methylation signature of chronic low-grade inflammation relevant to OP risk biology	Multicohort; DNAm–CRP signal for systemic inflammation	Wielscher et al. (2022)

## 3 The role of DNA methylation in OP

DNA methylation denotes the incorporation of a methyl group onto the cytosine nucleotide of a DNA strand, forming 5-methylcytosine. This process primarily relies on three types of DNA methyltransferases (DNMTs), namely DNMT1, DNMT3A, and DNMT3B. DNA methylation predominantly occurs in CpG islands, with high methylation generally associated with gene silencing, while low methylation may promote gene expression (Kim, 2025). In recent years, a substantial body of research has demonstrated that DNA methylation plays a pivotal role in the pathogenesis and progression of OP, with its underlying mechanisms primarily involving two aspects. Initially, DNA methylation regulates key genes and signaling pathways associated with bone metabolism, thereby influencing the functional equilibrium between OB and OC. Secondly, aberrant DNA methylation in immune cells may disrupt homeostasis within the bone-immune system. These findings not only enhance our understanding of the molecular basis of OP but also underscore the critical role of DNA methylation in the cross-talk between skeletal and immune systems.

### 3.1 DNA methylation regulates key genes and signaling pathways involved in bone metabolism

Genome-wide DNA methylation study in PMO women revealed 13 differently methylated genes. Notably, GNA11 is associated with calcium ion signaling and the cGMP-PKG signaling route, PRKCZ is engaged in endocytosis and the Rap1 signaling pathway, and SCD participates in the AMPK signaling network. These findings indicate that DNA methylation may play a role in the pathogenesis of OP by regulating many genes and their corresponding signaling pathways (Zhou et al., 2020). The interaction of Wnt ligands with their receptors in the Wnt/β-catenin signaling pathway facilitates OB differentiation. A study by García-Ibarbia et al. revealed that, in contrast to patients with osteoarthritis, individuals with OP hip fractures display markedly diminished Wnt pathway activity and lower nuclear β-catenin levels in OB. The disparity is ascribed to the varying methylation levels of genes associated with the Wnt signaling pathway in the bone tissues of the two groups (García-Ibarbia et al., 2013). Wu et al. discovered that the promoter of the Wnt receptor Frizzled1 (FZD1) gene exhibited aberrant hypermethylation in the mesenchymal stem cells (MSCs) of patients with femoral head necrosis, resulting in diminished expression of FZD1 and downregulation of the Wnt/β-catenin signaling pathway (Wu et al., 2019). In addition, the RANK/RANKL/OPG signaling pathway serves as the principal regulatory mechanism controlling OC differentiation and function. Recent investigations have shown that DNA methylation significantly influences the OPG-RANKL system in both murine models and human bone tissue. In mouse ST2 cells, RANKL expression is tightly associated with the methylation of its promoter. The administration of DNMT inhibitors (DNMTi) markedly enhances RANKL expression and the OC formation (Kitazawa and Kitazawa, 2007). In the bone tissue of individuals with OP fractures, the promoter of RANKL is in a state of low methylation, whereas the promoter of OPG is heavily methylated, resulting in elevated RANKL expression and reduced OPG levels. This indicates that the dysregulation of the OPG-RANKL pathway may precipitate OP via DNA methylation (Wang et al., 2018). In summary, DNA methylation is fundamental to OP by modulating the classical signaling pathway.

OBs originate from MSCs. During the differentiation of MSCs into OBs and subsequently into osteocytes, significant alterations in gene promoter methylation transpire (Valenti et al., 2016). RUNX2 and OSX are unique transcription factors essential for OB differentiation and bone formation. Research indicates that the expression of RUNX2 and its downstream target OSX is elevated, although the methylation level in their promoter regions are markedly reduced (Zhang et al., 2011). Similarly, Reppe et al.'s research shows that SOST protein, as a negative regulator of bone formation, inhibits the activation of the Wnt/β-catenin signaling pathway by binding to LRP5/6, thereby suppressing bone formation. In OP patients, heightened methylation of the SOST diminishes blood SOST levels, hence mitigating the suppression of Wnt signaling and promoting bone formation (Reppe et al., 2015). Simultaneously, Marofi et al. discovered that DNA methylation in the promoter regions of the ZBTB16 and Twist1 genes might be one of the key mechanisms controlling MSC differentiation into OBs (Marofi et al., 2019). Furthermore, Nomura et al. also identified that the activity of DNMT3A and DNMT3B was markedly elevated during MSC chondrogenic differentiation, and overexpression of DNMT3A substantially augmented the chondrogenic potential of MSCs (Nomura et al., 2019). These studies underscore the pivotal role of DNA methylation in regulating the expression of key OB genes.

OCs originate from the monocyte lineage and are chiefly accountable for the resorption of bone matrix. The gene expression of nuclear factor of activated T-cells 1 (NFATc1), a crucial transcription factor that promotes OC differentiation, is substantially modulated by DNA methylation. Studies have shown that diminishing the methylation level of NFATc1 can augment OC activity and accelerate bone resorption (Kurotaki et al., 2020). OC differentiation is similarly contingent upon M-CSF/RANKL signaling. Additionally, RANKL is secreted by OB, and thus the OPG/RANKL/RANK signaling pathway mediates the interaction between OB and OC (Boyce et al., 2023). In the hyperhomocysteinemia OP model, overexpression of DNMT1 results in hypermethylation of the OPG promoter, inhibiting OPG transcription and enhancing RANKL expression, consequently facilitating OC formation and bone loss (Behera et al., 2018). Nishikawa et al. conducted a study demonstrating that S-adenosylmethionine (SAM) influences OC differentiation via DNMT3A. Among them, SAM promotes OC formation by inhibiting anti-osteoclast factors, including RANKL and IRF8. In a postmenopausal OP model, the administration of DNMT3A inhibitor (theaflavin-3,3′-digallate) diminished the quantity of activated OC and augmented bone mass, thereby aiding in the prevention of bone loss in OP (Nishikawa et al., 2015). All evidence together substantiates that DNA methylation regulates OC differentiation and function via multifaceted methods. The major DNA methylation regulators that govern osteoblast, osteoclast, and mesenchymal stem cell fate decisions in OP are summarized in Table 2.

**TABLE 2:** Methylation regulators driving bone cell fate (osteoclast/osteoblast/BMSC).

Study (year)	Species/cells	Methylation target (type)	Direction and mechanism	Main bone effect	Refs
Nishikawa et al., 2015 (Nat Med)	Mouse; OC precursors	IRF8 (DNA; via DNMT3A/SAM)	RANKL ↑ oxidative metabolism → ↑SAM fuels DNMT3A → IRF8 promoter methylation → OC program stabilized	Promotes osteoclastogenesis and bone loss	Nishikawa et al. (2015)
Reppe et al., 2015 (JBMR)	Human bone	SOST (DNA)	Higher promoter methylation associates with ↑ serum sclerostin and fragility fractures	Predicts fracture risk	Reppe et al. (2015)
Li et al., 2018 (Bone)	Rat disuse OP; UMR-106 cells	H19 (DNA; via DNMT1)	DNMT1 overexpression → H19 hypermethylation; 5-aza/decitabine rescues	Impaired osteogenesis; rescue by DNMT inhibition	Li et al. (2018)
Morris et al., 2017 (JBMR)	Human blood (EWAS)	Multiple CpGs (DNA)	EWAS of BMD; limited large-effect blood CpGs	Suggests tissue-specific methylation for bone traits	Morris et al. (2017)
Yalaev et al., 2024 (IJMS)	Human blood	RUNX2 (DNA)	RUNX2 promoter hypomethylation (men > women) in primary OP	Candidate diagnostic marker	Yalaev et al. (2024)
Yang et al., 2023 (Cell Death Dis)	Mouse and human cells	NFATc1 (RNA m6A; METTL14/YTHDF2)	Exosome-delivered METTL14 ↑ m6A at NFATc1 (4249A) → mRNA decay	Inhibits osteoclast resorption, preserves bone	Yang et al. (2023)

### 3.2 The role of immune cell DNA methylation in OP

DNA methylation is integral to the entire process of immune cell differentiation. There remains an absence of causal evidence regarding whether specific methylation events directly influence the immune system (Calle-Fabregat et al., 2020). To tackle this issue, researchers chose a model of human B leukemia cells undergoing differentiation into macrophages. Through whole-genome bisulfite sequencing (WGBS), researchers revealed a significant negative connection between demethylation in the IL1RN promoter region and gene expression. The CRISPR-dCas9-TET1 can be employed to target and induce demethylation in this area, hence facilitating the overexpression of IL1RN in B cells. In contrast, utilizing dCas9-DNMT3A to sustain an elevated methylation state in the promoter obstructs the production of essential myeloid differentiation markers such as CD14 and CD11b. This study advances the understanding of CRISPR-mediated DNA methylation in regulating immune cells and inflammatory responses (Valcárcel et al., 2025).

Growing evidence indicates that DNA methylation impacts immune system function via many pathways. For example, in patients with immune thrombocytopenia, the overall DNA methylation levels of CD4^+^ T cells are diminished, with particularly pronounced abnormalities in the methylation of the promoter regions of key immune regulatory genes such as PD-1 and FOXP3, resulting in functional impairments in regulatory T cells (Treg) and compromising self-tolerance (Wang et al., 2024b). Clonal hematopoiesis (CH) is also related to immune dysregulation and is involved with several disorders (cardiovascular diseases, OP, etc.). The research demonstrates that mutations in genes such as TET2 and DNMT3A in myeloid cells can enhance the expression of chemokines and over-activate the inflammasome, leading to immune imbalance and chronic inflammation in CH (Belizaire et al., 2023). Specifically in the OP process, aberrant methylation of immune cells can directly influence the formation of OC and bone resorption. In the OVX mouse model, the aberrant increase of DNMT3A in bone marrow-derived macrophages (BMMs) results in a reduction in FoxO3 expression. FoxO3 regulates ROS, and its diminished expression facilitates excessive OC formation, increases bone resorption, and accelerates bone loss (Zhang et al., 2025). In addition, the NF-κB pathway, as a key inflammatory pathway, is affected by DNA methylation. Aberrant functions of DNMT3A and TET2 can influence the magnitude of the inflammatory response through the NF-κB pathway (Wang et al., 2024a). In conclusion, although existing studies have indicated the significant role of immune cell DNA methylation in OP, the specific mechanism of this effect still requires more comprehensive experimental evidence to be clarified.

## 4 DNA methylation signatures as biomarkers and therapeutic targets in OP

DNA methylation patterns are emerging as both actionable treatment targets and promising biomarkers in OP. In addition to conventional pharmacotherapies that regulate bone turnover, inhibition of DNMT—through small molecules, RNA-based approaches, or naturally derived compounds—has shown effectiveness in preclinical models. Concurrent progress in epigenomic profiling has identified methylation patterns in bone-resorption and bone-formation genes, so peripheral blood methylation profiles may act as proxies for bone tissue. These biomarkers could facilitate minimally invasive risk classification, early diagnosis, and therapy monitoring, thereby enhancing precision medicine approaches. Combining targeted epigenetic regulation with biomarker-driven patient selection has the potential to enhance therapy efficacy, minimize side effects, and customize OP management.

### 4.1 Current pharmacotherapies for OP

A variety of pharmaceuticals are accessible for the management of OP. Various therapy regimens have been developed, grouped by their mechanism of action into bone resorption inhibitors, bone formation promoters, dual-action medicines, and medications with alternative mechanisms (LeBoff et al., 2022). Each category has been utilized alone, and in certain therapeutic trials, they have also been employed in conjunction.

Bone resorption inhibitors, including bisphosphonates (alendronate sodium, zoledronic acid, risedronate sodium, ibandronate sodium, minodronate), RANKL inhibitors (denosumab), selective estrogen receptor modulators (raloxifene), and calcitonin (salmon calcitonin) (Song et al., 2022). The majority of patients will commence treatment with bisphosphonates. If the treatment proves ineffective or the adverse effects are too severe, they may transition to RANKL inhibitors. Denosumab, by inhibiting the RANKL protein, can mitigate bone loss, decrease fractures risk, and is appropriate for individuals with renal impairments. Selective estrogen receptor modulators, in conjunction with various estrogen receptors types, impede bone resorption and are suitable for PMO women (Rani et al., 2023). Calcitonin, an essential calcium-regulating hormone, facilitates calcium deposition into bone and mitigates pain by decreasing prostaglandin synthesis, rendering it a favored choice for OP patients experiencing concurrent bone pain (Jain and Chatterjee, 2020). Bone formation promoters are primarily parathyroid hormone analogs (teriparatide), which promote new bone formation by preferentially stimulating the PTH1R/Wnt signaling pathway, and are typically employed for severe OP patients (Chen et al., 2021a). Dual-action drugs are primarily anti-sclerostin monoclonal antibodies (romosozumab), which function by simultaneously promoting bone formation and inhibiting bone resorption through targeted inhibition of SOST/LPR binding and blockade of the Wnt signaling pathway, rendering them appropriate for the management of severe OP (Marini et al., 2023). Agents of other mechanisms generally consist of active vitamin D and its analogs (e.g., alfacalcidol, calcitriol, eldecalcitol). These medicines demonstrate physiological activity independent of renal 1α-hydroxylation, making them appropriate for older patients, those with renal impairment, or individuals deficient in 1α-hydroxylase activity (Xiong et al., 2024). Nonetheless, current OP medications predominantly focus on the cells of the bone remodeling unit, and prolonged usage may overly inhibit bone metabolism, hence elevating the chance of atypical fractures. Consequently, therapy should be suspended following extended use to mitigate adverse effects (Fink et al., 2019).

### 4.2 Therapeutic potential of DNA methylation inhibitors in bone metabolism regulation

Numerous current therapies focus on enzymes that create or remove epigenetic modifications. Among these, DNMTi alter DNA methylation. Extensive study underscores the therapeutic efficacy of DNMTi in ameliorating OP. For instance, in a rat model of disuse osteoporosis, local injection of siRNA targeting DNMT1 (a gene-silencing form of DNMTi) effectively improved compromised femoral microstructure, offering direct evidence for the bone-protective effects of DNMT1 inhibition (Li et al., 2018). As a physiological intervention, exercise can suppress the increased DNMT1/3a/3b activity in OVX mice, and concurrently rectify the aberrant hypermethylation of the Nrf2 promoter, thus mitigating OP. This suggests that exercise may influence outcomes via a mechanism similar to that of DNMTi (Chen et al., 2021b). Naturally derived DNMTi exhibit unique benefits in traditional Chinese medicine for OP prevention and treatment. Diverse plant-derived active compounds (such as polyphenols, flavonoids, and alkaloids) have DNMTi properties. For example, glycyrrhizic acid can mitigate pulmonary fibrosis by blocking DNMT1/3A (Wei et al., 2022). Most recently, research has revealed that in diabetic osteoporosis (DOP), curcumin saponin VI (AVI) reduces bone loss by inhibiting the aberrant DNMT1/3A activation and the consequent hypermethylation of the glutathione peroxidase 4 (Gpx4) promoter, thus obstructing the ferroptosis pathway in OBs (Wei et al., 2024). Increasing data suggests that 5-aza-2′-deoxycytidine (5-AZA-dC, decitabine), the predominant DNMTi, promotes DNA demethylation and significantly influences the modulation of innate immune responses and anti-tumor capabilities (Gonda et al., 2020). Chen et al. established that Wnt10a is a crucial factor in the differentiation of mesenchymal cells into toward OBs. 5-Aza-dC can markedly impede adipogenesis and promote OB generation by demethylating the 5′region (rich in CpG sites) of the Wnt10a gene. This discovery suggests the utilization of conventional pharmacologic DNMTi, such as 5-AZA-dC, as a possible treatment option for people with concurrent obesity and OP (Chen et al., 2016). Zhou et al. conducted a study demonstrating that 5-AZA-dC enhances the expression and differentiation of osteogenic genes in MSC by modifying DNA methylation (Zhou et al., 2009). All findings collectively demonstrate that DNMTi show therapeutic effects in animal models, indicating that medications aimed at inhibiting DNA methylation are pivotal in managing the equilibrium bone metabolism. Representative preclinical and experimental interventions that modulate DNA methylation to preserve bone mass are presented in Table 3.

**TABLE 3:** Interventions that (de)regulate methylation and protect bone.

Intervention	Primary methylation target/change	Model and outcome	What it shows for osteoporosis	Refs
Aerobic running exercise	Demethylation of Nrf2 promoter; ↓DNMTs in femur	OVX mice + running; restored trabecular bone	Physiologic loading can re-open antioxidant defenses via DNA demethylation to counter bone loss	Chen et al. (2021b)
siRNA against DNMT1 (local)	↓H19 promoter 5mC; ↑ERK signaling	Disuse osteoporosis (rat) + DNMT1 knockdown; ↑osteogenesis	Direct DNMT1 suppression rescues osteogenic pathways in unloading-induced bone loss	Li et al. (2018)
5-Aza-2′-deoxycytidine (decitabine)	Global DNA demethylation; Wnt10a promoter activation	MSCs/3T3-L1; shift adipogenesis→osteoblastogenesis	DNMT inhibition biases lineage toward osteoblasts via Wnt signaling	Chen et al. (2016)
Exosome-delivered METTL14 (m6A)	↑m6A on NFATc1 (A4249); YTHDF-mediated repression	Osteoclast models + targeted exosomes; ↓bone resorption	RNA methylation editing can restrain osteoclastogenesis and preserve bone	Yang et al. (2023)
S-adenosyl-methionine (SAM) pathway (mechanistic)	DNMT3A couples to SAM production to silence Irf8	OC differentiation; metabolic–epigenetic coupling	Metabolic supply of methyl groups drives OC commitment via DNMT3A	Nishikawa et al. (2015)
Hydrogen sulfide (H_2_S) donors	Epigenetic modulation of OPG/RANKL axis	HHcy mouse model; mitigated bone loss	Gasotransmitter counters methylation-linked osteoclast activation	Behera et al. (2018)
Vitamin C (ascorbate)	TET co-factor; ↑5hmC and histone demethylation at osteogenic loci	OB lineage + mice; ↑bone mass/osteogenesis	Pharmacologic demethylation support enhances osteoblast programs *in vivo*	Thaler et al. (2022)
DNMT inhibitor (SGI-1027)/DNMT suppression	Relief of GPX4 promoter hypermethylation; anti-ferroptosis in OBs	OVX mice; prevents osteoblastic ferroptosis and bone loss	Targeting DNMT-driven GPX4 silencing protects bone-forming cells	Wei et al. (2024)
α-Ketoglutarate (αKG) supplementation	Co-factor for TET/HDMs; ↓H3K9me3/H3K27me3	Aged mice; ↑bone mass, ↑MSC osteogenesis	Metabolic support of demethylases rejuvenates bone anabolism	Wang et al. (2020)
DNMT3A gain-of-function in OBs	Methylation of PPARγ promoter; shifts OB metabolism	Postmenopausal OP models; ↑OB differentiation	Context matters: DNMT3A can be pro-osteogenic in osteoblast lineage	Lu et al. (2022)

### 4.3 DNA methylation biomarkers for precision medicine in OP

Bone turnover markers (BTMs) quantify specific biochemical substances in blood or urine to reflect the activity of OB-mediated bone formation and OC-mediated bone resorption, providing a dynamic evaluation of skeletal metabolic balance. When it comes to BTM, on one hand, studies have shown that BTM can also predict fracture risk and the pace of bone loss. On the other hand, the biochemical BTM may assist researchers in elucidating the mechanism of action of medicinal medications during clinical trials (Williams et al., 2025). Nonetheless, the considerable individual variability across patients and the inadequate standardization of the majority of detection and analysis protocols have emerged as constraints of BTM in clinical practice (Hlaing and Compston, 2014). Despite being derived from the disintegration and synthesis of bone tissue, BTMs do not exhibit specificity for distinct bone tissue sites, failing to differentiate the bone turnover conditions of periosteum, cortical bone, and cancellous bone. In the initial phase of an illness or during overlapping oscillations in other physiological states, alterations in BTM may not be evident (Williams et al., 2025). Conversely, alterations in DNA methylation may transpire before the onset of disease or during its initial phases. A recent study conducted DNA methylation profiling on bone and whole blood samples obtained from 12 patients receiving surgical treatment for hip fractures, indicating that alleviating aberrant DNA methylation may decrease mortality linked to hip fractures (Lane and Witayakom, 2023). Moreover, DNA methylation provides comprehensive insights into the regulation of gene expression and cellular functional states in OP, hence augmenting our comprehension of disease pathogenesis and development. This epigenetic mechanism may serve as a valuable foundation for the development of personalized therapeutic strategies. The future combination of these methodologies promises to provide more comprehensive solutions for OP risk prediction and clinical management.

Recent advancements in molecular biology have recognized aberrant DNA methylation patterns as potential biomarkers for various diseases. DNA methylation biomarkers have substantial relationships with disease prognosis, activity, and therapeutic responsiveness in early-stage inflammatory arthritis, indicating that DNA methylation signatures of immune cell subsets may serve as clinically valuable biomarkers (Declerck and Vanden Berghe, 2019). For example, in the peripheral blood of IBD, the methylation risk assessment score of the C-reactive protein (CRP) gene markedly elevates, and the result analysis indicates a positive correlation with the disease activity index (Wielscher et al., 2022). Peripheral blood analyses of infants with severe bronchiolitis indicate that differentially methylated CpG sites are linked to the functional states of specific immune cell subsets (e.g., helper T cells, neutrophils), and influence disease progression by regulating lung tissue-specific gene expression and IL-1 signaling pathway activation (Zhu et al., 2023). Due to the difficulties in acquiring bone specimens, the exploration of differential DNA methylation indicators derived from peripheral blood has emerged as a crucial research focus for OP assessment. Ebrahimi et al. validated this approach by demonstrating substantial epigenomic concordance between bone and blood. Approximately 28,549 CpG sites exhibited consistent methylation patterns across both tissues, with 33%–49% connected with bone phenotypes and enriched in bone regulatory pathways, suggesting that peripheral blood captures bone-related methylation signatures (Zhao et al., 2025). The above research will advance future individualized treatment options by investigating if DNA methylation markers in the blood can function preliminary tools for OP diagnosis or risk assessment, aiding doctors in managing populations with a high occurrence rate. Candidate DNA methylation biomarkers with potential utility for OP diagnosis, prognosis, and risk stratification are listed in Table 4.

**TABLE 4:** Candidate methylation biomarkers (discovery → validation pipeline).

Locus/Signature	Specimen	Clinical readout	Study design and size	Translational status	Refs
SOST promoter methylation ↔ bone SOST mRNA ↔ serum sclerostin	Iliac bone + serum	Strong correlation with fracture risk in postmenopausal women	Case–control, n ≈ 200	Mechanistic + prognostic lead; requires non-invasive surrogate	Reppe et al. (2015)
EWAS of BMD (multi-CpG panel)	Whole blood	CpGs associated with DXA BMD	Population EWAS	Discovery stage for risk scoring	Morris et al. (2017)
Whole-blood DNAm “signature” in PMOP	Whole blood	OP vs control classification	Case–control	Candidate panel; needs external validation	Cheishvili et al. (2018)
RUNX2 promoter hypomethylation	Whole blood	Lower RUNX2 methylation in primary OP (men and women)	Cross-sectional	Promising single-locus marker; easy assay	Yalaev et al. (2024)
Chronic low-grade inflammation DNAm score (CRP-related)	Whole blood	Systemic inflammaging (OP comorbidity axis)	Large multi-cohort	Useful covariate to adjust OP biomarker models	Wielscher et al. (2022)
Bone–blood concordance panel	Matched bone and blood	28,549 CpGs with concordant methylation; enrichment in EN1/ESR1/WNT16/RANKL	Matched-tissue study	Identifies CpGs feasible for blood-based OP tests	Ebrahimi et al. (2021)
Bone vs blood differential methylome (postmenopausal women)	Bone and blood	Tissue-specific CpGs linked to BMD	Observational	Guides selection of blood-surrogate CpGs	Reppe et al. (2017)

The clinical efficacy of DNA methylation markers for screening and monitoring treatment response in OP patients is still ambiguous. The comprehensive DNA methylation patterns in bone tissue and peripheral blood were investigated utilizing chip technology, in an attempt to identify methylation indicators for the early diagnosis of OP. Generally speaking, DNA methylation patterns are typically regarded as tissue-specific. Reppe et al. examined 2,529 CpGs in the top 100 genes most substantially correlated with bone mineral density (BMD) and discovered that the methylation levels of 63 CpGs differed significantly different between healthy women and OP women. Notably, 13 of the 63 CpGs exhibited a connection with BMD in the bloodstream. In the comparison of high BMD with low BMD, the directional change for 12 of the markers (except for cg12876517 in UGP2) aligned with that observed in bone, indicating analogous regulation mechanisms in bone and blood are similar (Reppe et al., 2017). Cheishvili et al. examined the genome-wide DNA methylation profiles of peripheral blood samples from 22 healthy women and 22 PMO women, discovering 77 substantially differently methylated sites in the PMO group. Among them, the DNA methylation patterns of five significant genes exhibit great sensitivity and specificity in the early prediction of OP and are closely associated with BMD (Cheishvili et al., 2018). Contrary to the above results, Fernandez-Rebollo et al. compared the whole-genome DNA methylation profiles of peripheral blood from 16 non-OP patients and 32 primary OP patients, but did not find any significant abnormal methylation at any CpG site in OP patients (Fernandez-Rebollo et al., 2018). In conclusion, limited research exists about the potential of DNA methylation patterns in peripheral blood can serve as biomarkers for OP, and the findings are incongruous. Therefore, the clinical viability of employing DNA methylation biomarkers for the diagnosis of OP is very problematic.

## 5 Conclusion

**FIGURE 2:**
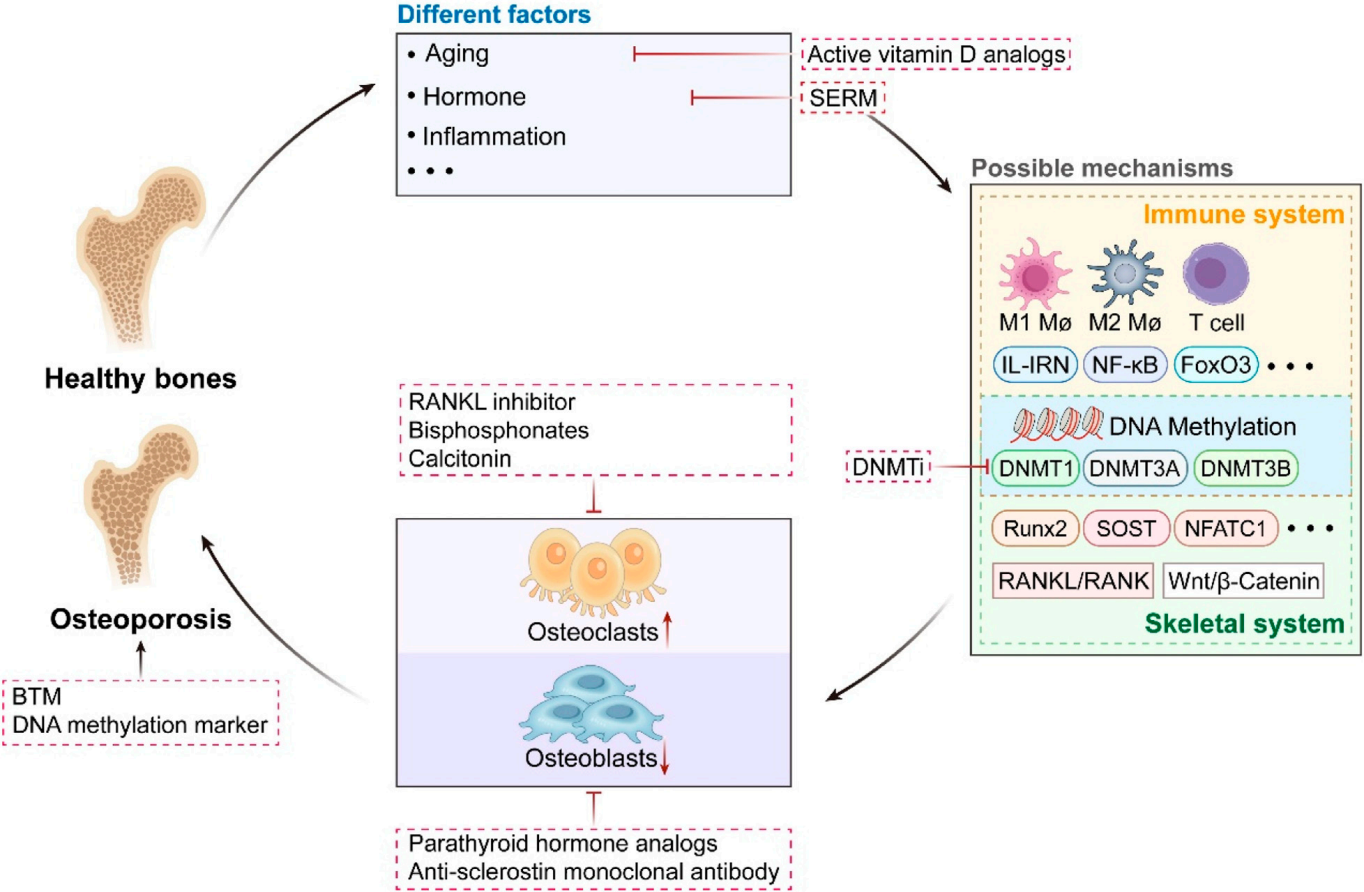
The Triad of Osteoporosis: Pathophysiology, Molecular Pathways, and Treatment Approaches. From healthy bones to the development of osteoporosis. It integrates various factors: aging, hormonal abnormalities, inflammation, etc. It involves multi-layered mechanisms: from immune-bone interaction regulation to epigenetic regulation mediated by DNA methylation, analyzing the molecular mechanisms of bone metabolism disorders. Specifically, DNA methylation mediated by DNMT1/3A/3B exerts dual regulatory effects: on one hand, it targets and modulates key bone metabolism-related genes such as Runx2, SOST, and NFATc1, as well as signaling pathways including RANKL/RANK and Wnt/β-Catenin; on the other hand, it influences immune-osseous signaling pathways involving M1/M2 macrophages, T cells, IL-1RN, and NF-κB. Both regulatory effects ultimately drive the imbalance between bone resorption and bone formation. It covers multiple therapeutic strategies: various existing treatments, the potential use of DNMTi. And correlates bone turnover markers (BTM) with the diagnostic value of DNA methylation biomarkers.

OP illustrates the complex interaction between skeletal and immune systems, wherein chronic low-grade inflammation and immune dysfunction expedite bone loss by favoring the balance toward OC-mediated resorption over OB- mediated creation. Advances in osteoimmunology has demonstrated that T cells, B cells, macrophages, neutrophils, and dendritic cells facilitate disease progression through cytokine networks including RANKL/OPG, Wnt, and IL-17 pathways. Significantly, these immune-mediated effects are not fixed but are dynamically influenced by epigenetic mechanisms, among which DNA methylation emerges as a pivotal regulator. Aberrant DNA methylation influences genes critical for bone cell differentiation and function—such as RUNX2, SOST, NFATc1, and FoxO3—while simultaneously reconfiguring immune cell phenotypes and inflammatory pathways, including NF-κB signaling and redox homeostasis. DNA methylation exerts a dual influence on skeletal cells and immunological effectors, situating it at the intersection of bone metabolism and immune regulation. Preclinical investigations indicate that the inhibition of DNMT or the modulation of methyl donor availability can reestablish osteoimmune balance. A substantial accumulation evidence suggests that DNMTi can proficiently reverse aberrant DNA methylation in OP (Figure 2). Both pharmacologic agents (e.g., 5-aza-2′-deoxycytidine, theaflavin-3,3′-digallate) and physiological interventions (e.g., exercise, nutrient supplementation) have demonstrated potential in reversing abnormal methylation signatures.

In addition to therapeutic targeting, DNA methylation holds potential as a biomarker platform for precision medicine in OP. Methylation signatures collected from peripheral blood exhibit significant concordance with bone tissue profiles and are linked to fracture risk, bone mineral density, and systemic inflammatory burden. Such epigenetic indicators may enable early diagnosis, fracture risk categorization, and real-time assessment of treatment efficacy, especially in situations where bone biopsy is unfeasible. However, research in this field is still in its infancy. On the one hand, the differentially methylated genes identified by various studies are inconsistent. Although the methylation status of these genes is significantly associated with OP, the vast majority are not the well-known classic bone metabolism-related genes, which may be related to the genetic background differences among the study populations. On the other hand, the regulatory network of bone metabolism in OP patients may be more complex than currently understood. For instance, there is a close interaction between the skeletal system and the immune system, but abnormal DNA methylation in immune cells may not be directly detectable in OP patients. Consequently, it is necessary to conduct large-sample clinical case-control studies and biological experiments in different populations. The integration of methylation biomarkers with clinical data and imaging may facilitate more tailored, mechanism-based treatment. Future research must emphasize longitudinal and multi-omics studies to elucidate causative methylation alterations that instigate osteoimmune dysregulation, rather than only indicating downstream pathology. Single-cell and spatial epigenomics may further resolve cell type–specific methylation patterns inside the bone marrow niche. Furthermore, the therapeutic application of DNMTi and methylation-modifying nutraceuticals necessitates meticulous tuning to reconcile efficacy with long-term safety, considering the multifaceted roles of DNA methylation across many tissues.

The innovation of this review resides in the groundbreaking establishment of a systematic integration framework of the DNA methylation-bone immune regulation axis, which elucidates the intricate molecular association mechanism between chronic immune dysregulation and bone metabolic imbalance. It fills the gap of the traditional research which analyzed the three elements in isolation. Furthermore, it advances epigenetics from fundamental mechanisms to clinical applications in OP, distinctly highlighting its dual potential in dynamic biomarkers and reversible therapeutic targets. This advancement signifies the decoding and use of DNA methylation in the context of osteoimmunity as a prospective avenue for OP prevention, diagnosis, and therapy. By integrating molecular mechanisms with clinical practice, epigenetic techniques could transform OP care from mere symptom control to sustainable, focused restoration of skeletal health.

## Abbreviations

OP, osteoporosis; OB, osteoblast; OC, osteoclast; DNMT, DNA methyltransferase; DNMTi, DNMT inhibitors; OVX, ovariectomized; RA, rheumatoid arthritis; IBD, inflammatory bowel disease; BTM, Bone turnover marker; MSC, mesenchymal stem cells; CH, Clonal hematopoiesis; DCs, Dendritic cells; Bregs, Regulatory B cells; Th, Helper T cells; PMO, postmenopausal osteoporosis; 5-AZA-Dc, 5-aza-2′-deoxycytidine; BMD, bone mineral density.
